# 5,14-Diselena-3,4,15,16-tetra­aza­tri­cyclo[11.3.0.0^2,6^]hexa­deca-1(13),2(6),3,15-tetra­ene

**DOI:** 10.1107/S2414314626010953

**Published:** 2026-10-23

**Authors:** Dieter Schollmeyer, Heiner Detert

**Affiliations:** a University of Mainz, Department of Chemistry, Duesbergweg 10-14, 55099 Mainz, Germany; University of Aberdeen, United Kingdom

**Keywords:** crystal structure

## Abstract

The complete mol­ecule of the title compound, C_8_H_8_N_4_Se_2_, is generated by a crystallographic twofold axis: the almost planar 1,2,3-selena­diazole ring and its symmetry-generated partner are annulated to a cyclo­deca-1,3-diene ring. Their dihedral angle amounts to 88.4 (2)° and the conformation of the hexa­methyl­ene chain is mostly staggered (*gauche*) except for a near-*anti* arrangement in the middle.

## Abstract 2

The complete mol­ecule of the title compound is generated by a crystallographic twofold axis: the almost planar 1,2,3-selena­diazole ring and its symmetry-generated partner are annulated to a cyclo­deca-1,3-diene ring. Their dihedral angle amounts to 88.4 (2)° and the conformation of the hexa­methyl­ene chain is mostly staggered (*gauche*) except for a near-*anti* arrangement in the middle.

## Graphical abstract

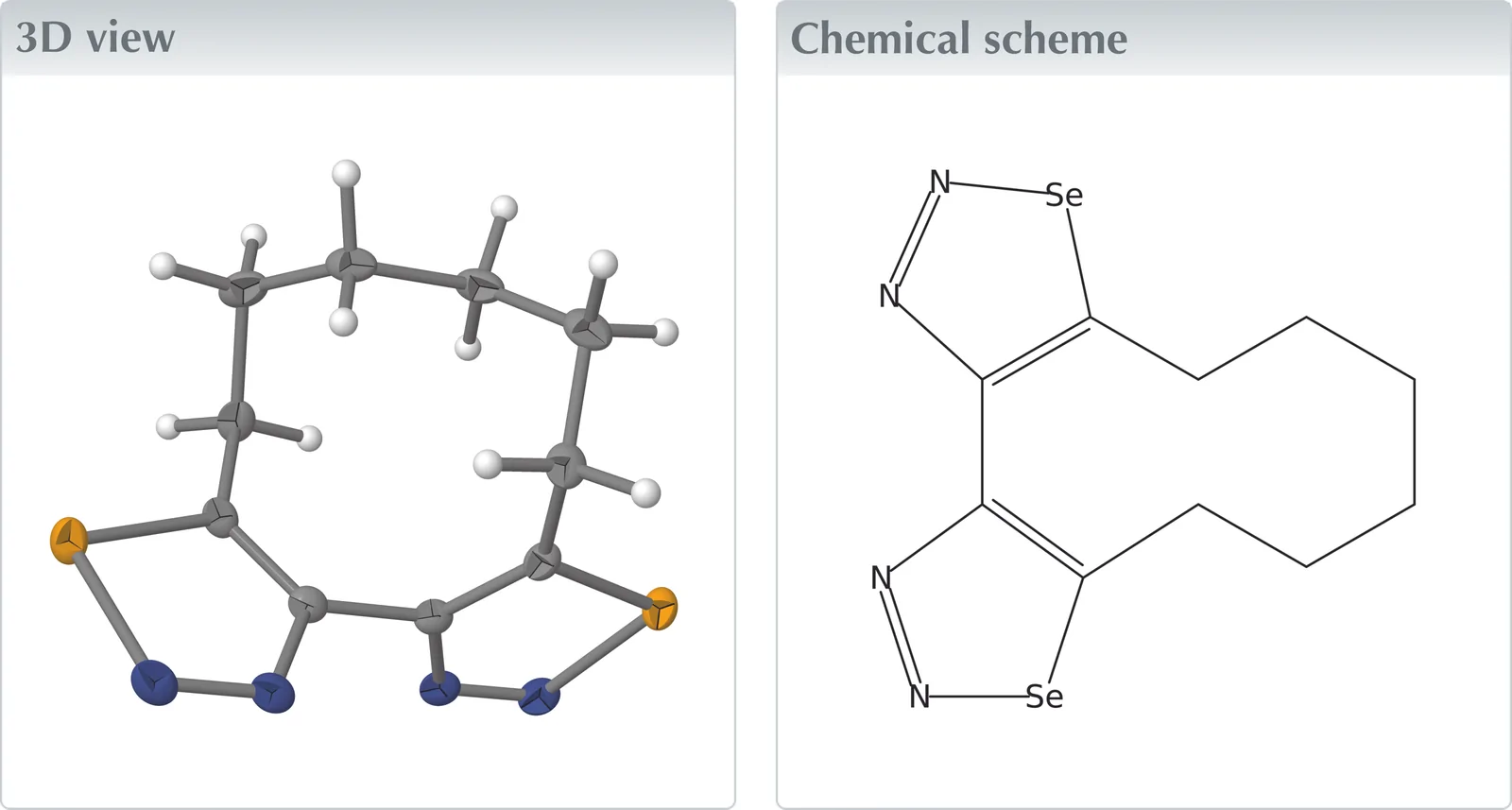

## Structure description

The title compound,*C*_8_H_8_N_4_Se_2_ (**I**), was prepared in a project focusing on medium-sized cyclo­alkynes with additional sterically demanding groups (Bissinger *et al.* 1988 ▸; Detert *et al.* 1994 ▸; Detert & Meier, 1997 ▸). Bis-1,2,3-selana­diazo­les are important sources for medium-sized cyclo­alkadiynes (Gleiter *et al.* 1988 ▸) and the structures of three bis­selena­diazolo-cyclo­octa­dienes have recently been reported (Detert & Schollmeyer, 2020 ▸, 2026 ▸, Schollmeyer & Detert, 2025 ▸). Compound (**I**) is thermally unstable and in the presence of tetra­phenyl cyclo­penta­dienone, the formal mono-trapping product of cyclo­deca-1,3-diyne is formed (42%, m.p. 529 K).

The complete mol­ecule of (**I**) (Fig. 1 ▸) is generated by crystallographic twofold symmetry with the rotation axis passing through the mid-points of the C1—C1^i^ and C8—C8^i^ bonds [symmetry code: (i) 1 − *x*, *y*, 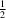 − *z*] with a dihedral angle between the 1,2,3-selena­diazole ring and its symmetry-generated partner of 88.4 (2)°. This angle is more than twice as large as in the smaller eight-ring homologue (43.6°, Detert & Schollmeyer, 2020 ▸). The C1/N2/N3/Se4/C5 selena­diazole ring is planar to within 0.05 (4) Å and the N2—C1—C1^i^—N2^i^ torsion angle is 91.8 (4)°. The hexa­methyl­ene tether shows a nearly perfect staggered conformation with torsion angles of *ca* 60°; only the central C7—C8—C8^i^—C7^i^ torsion angle of −149.8 (4)° indicates some Pitzer strain. Within the ortho­rhom­bic crystal, the twofold symmetry axis parallel to the *b*-axis and a twofold screw axis along the *c*-axis pack the mol­ecules effectively (Fig. 2 ▸).

## Synthesis and crystallization

The title compound was prepared from sebacoic acid *via* esterification with ethanol, an acyl­oin condensation according to a combination of the Prelog and Rühlmann procedures (Prelog *et al.* 1947 ▸; Rühlmann 1971 ▸), oxidation with copper acetate (Blomquist & Goldstein 1956 ▸), formation of the semicarbazone and reaction with selenium dioxide. The semicarbazone (8.5 g, m.p. 556 K) was suspended in dioxane (70 ml), and selenium dioxide (26.4 g) and water (15 ml) were added and the mixture was stirred at 318 K for 4 days. Filtration, removal of 50 ml dioxane, addition of 50 ml of ethyl acetate, washing of the organic layer with brine, sodium bicarbonate solution, and brine was followed by drying over sodium sulfate and chromatography on silica gel with toluene/ethyl acetate 7/3 yielded 3.5 g (34%) of hexa­gonal, pinkish crystals with m.p. 398–400 K (dec.). Single crystals of (**I**) were grown by slow evaporation of a solution in toluene. Spectroscopic data: (assignment of signals follows IUPAC nomenclature): ^1^H-NMR (400 MHz, CDCl_3_): δ = 3.15, 2.90 (2 × *dt*, 2 × 2H, *J* = 15 Hz; *J*′= 5 Hz, fast exchange of H—CH’-7,12 and HC—H′-7,12), 1.65 (*m*, 4 H, H_2_C-8, 12), 1.35, 0.75 (2 × *bs*, 2 × 2 H, fast exchange of H—CH’-9,10 and HC—H′-9,10); ^13^C-NMR (100 MHz, CDCl_3_): δ = 168.5, 152.3 (C-1,2,6,13), 30.0 (C-7, 12,), 24.8, 20.0 (C-8,9,10,11); ^77^Se-NMR (73 MHz, CDCl_3_, SeO_2_/D_2_O as reference): δ = 213.4; UV(EtOH): λ (log ɛ) = 210 (4.10), 228 (4.16), 286 nm (3.42), MS (FD): 348 (*M*^+.^, Se_2_-isotope pattern), 320 (*M*^+.^—N_2_, Se_2_-isotope pattern), 240 (*M*^+.^—N_2_—Se, Se-isotope pattern); MS (EI): 292 (8%, *M*^+.^ − 2 N_2_, Se_2_-isotope pattern), 212 (5%, *M*^+.^-2 N_2_– Se) 183 (50%), 129 (77%), 91 (100%); IR (KBr): ν = 2920, 2840, 1502, 1448, 1101, 1055, 860, 842 cm^−1^

## Refinement

Crystal data, data collection and structure refinement details are summarized in Table 1 ▸.

## Supplementary Material

Crystal structure: contains datablock(s) I, global. DOI: 10.1107/S2414314626010953/hb4574sup1.cif

Structure factors: contains datablock(s) I. DOI: 10.1107/S2414314626010953/hb4574Isup2.hkl

Supporting information file. DOI: 10.1107/S2414314626010953/hb4574Isup3.cml

CCDC reference: 2596883

Additional supporting information:  crystallographic information; 3D view; checkCIF report

## Figures and Tables

**Figure 1:**
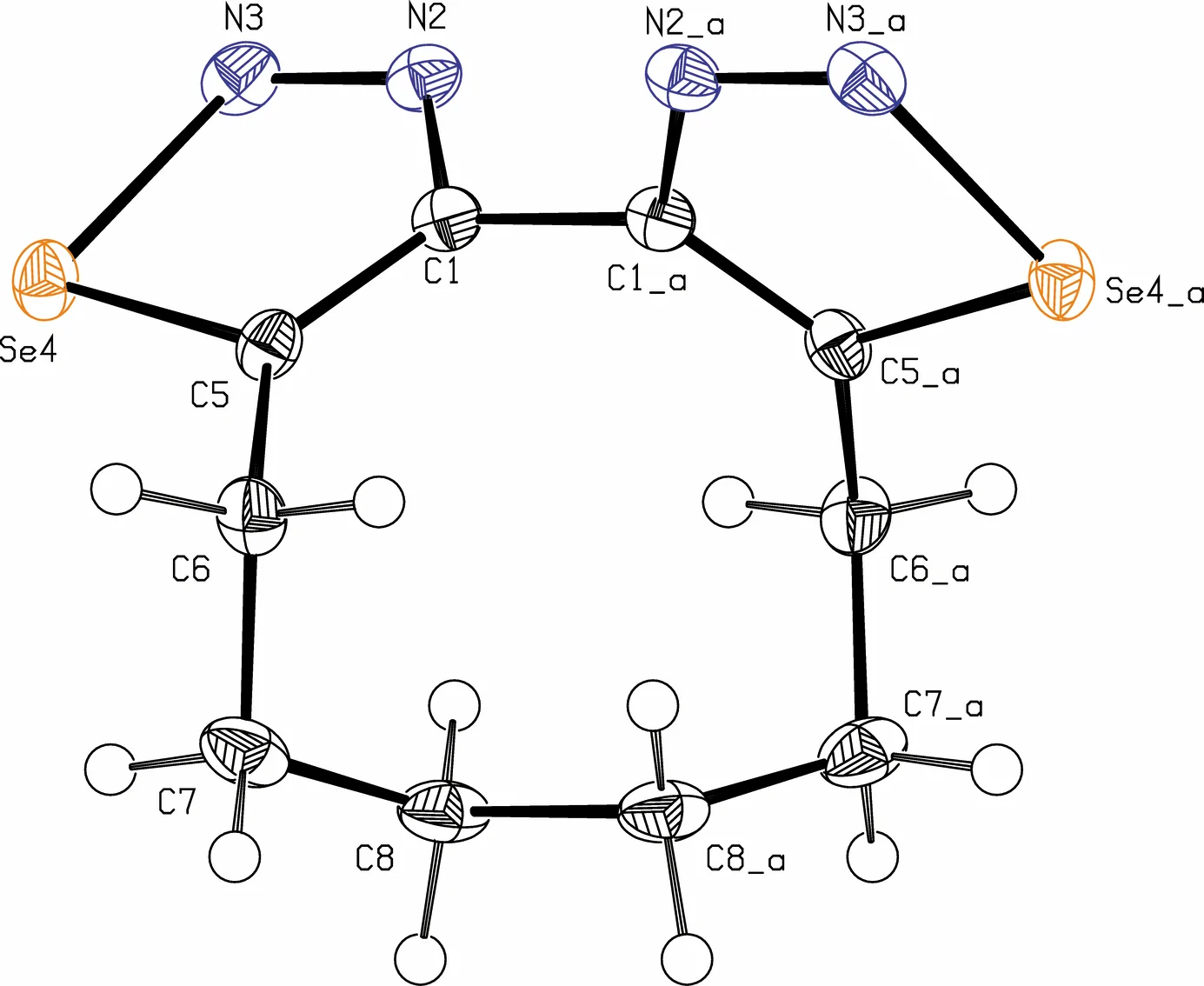
The mol­ecular structure of (**I**) with displacement ellipsoids drawn at the 50% probability level. Atoms with suffix _a are generated by the symmetry operation 1 − *x*, *y*, 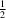 − *z*.

**Figure 2:**
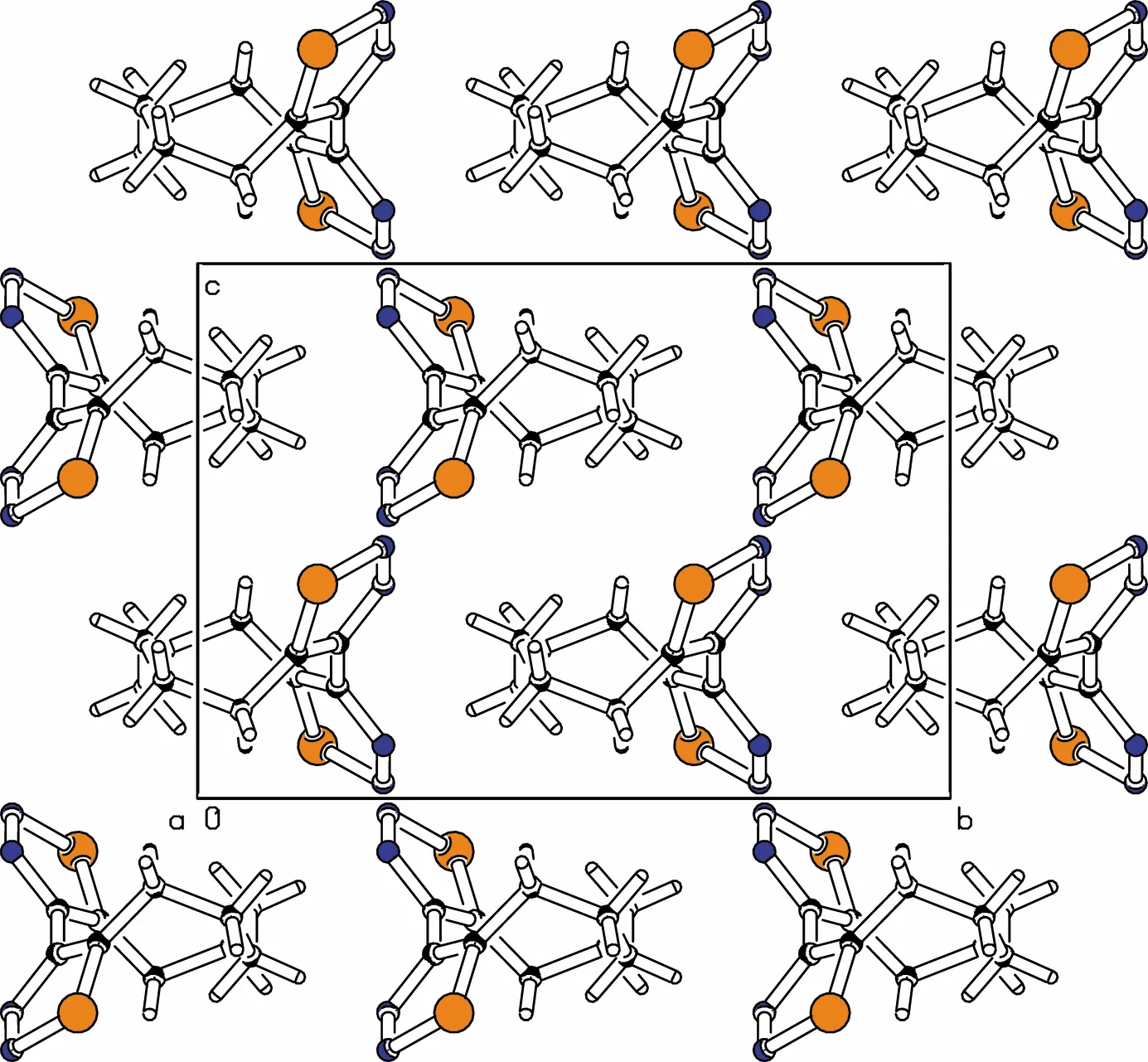
Part of the packing diagram of (**I**) viewed along the *a*-axis direction.

**Table 1:** Experimental details

Crystal data	
Chemical formula	C_10_H_12_N_4_Se_2_
*M*_r_	346.16
Crystal system, space group	Orthorhombic, *C*222_1_
Temperature (K)	120
*a*, *b*, *c* (Å)	8.4957 (7), 14.0651 (10), 9.9897 (6)
*V* (Å^3^)	1193.70 (15)
*Z*	4
Radiation type	Mo *K*α
μ (mm^−1^)	6.17
Crystal size (mm)	0.37 × 0.26 × 0.23

Data collection	
Diffractometer	Stoe IPDS 2T
Absorption correction	Integration
*T*_min_, *T*_max_	0.230, 0.329
No. of measured, independent and observed [*I* > 2σ(*I*)] reflections	1663, 1254, 1224
*R*_int_	0.014
(sin θ/λ)_max_ (Å^−1^)	0.658

Refinement	
*R*[*F*^2^ > 2σ(*F*^2^)], *wR*(*F*^2^), *S*	0.024, 0.060, 1.15
No. of reflections	1254
No. of parameters	73
H-atom treatment	H-atom parameters constrained
Δρ_max_, Δρ_min_ (e Å^−3^)	0.48, −0.31
Absolute structure	Classical Flack method preferred over Parsons because s.u. lower
Absolute structure parameter	0.08 (3)

Computer programs: *X-AREA WinXpose*, *X-AREA Recipe* and *X-AREA Integrate* (Stoe & Cie, 2020 ▸), *SHELXT2014* (Sheldrick, 2015*a* ▸), *SHELXL2019/2* (Sheldrick, 2015*b* ▸) and *PLATON* (Spek, 2009 ▸).

## full crystallographic data

### 5,14-Diselena-3,4,15,16-tetraazatricyclo[11.3.0.02,6]hexadeca-1(13),2(6),3,15-tetraene Crystal data

**Table:**

C_10_H_12_N_4_Se_2_	*D*_x_ = 1.926 Mg m^−^^3^
*M**_r_* = 346.16	Mo *K*α radiation, λ = 0.71073 Å
Orthorhombic, *C*222_1_	Cell parameters from 3676 reflections
*a* = 8.4957 (7) Å	θ = 2.8–28.3°
*b* = 14.0651 (10) Å	µ = 6.17 mm^−^^1^
*c* = 9.9897 (6) Å	*T* = 120 K
*V* = 1193.70 (15) Å^3^	Block, colorless
*Z* = 4	0.37 × 0.26 × 0.23 mm
*F*(000) = 672	

### 5,14-Diselena-3,4,15,16-tetraazatricyclo[11.3.0.02,6]hexadeca-1(13),2(6),3,15-tetraene Data collection

**Table:**

Stoe IPDS 2T diffractometer	1254 independent reflections
Radiation source: sealed tube	1224 reflections with *I* > 2σ(*I*)
Detector resolution: 6.67 pixels mm^-1^	*R*_int_ = 0.014
rotation method, ω scans	θ_max_ = 27.9°, θ_min_ = 2.8°
Absorption correction: integration	*h* = −11→10
*T*_min_ = 0.230, *T*_max_ = 0.329	*k* = −12→18
1663 measured reflections	*l* = −13→11

### 5,14-Diselena-3,4,15,16-tetraazatricyclo[11.3.0.02,6]hexadeca-1(13),2(6),3,15-tetraene Refinement

**Table:**

Refinement on *F*^2^	Hydrogen site location: inferred from neighbouring sites
Least-squares matrix: full	H-atom parameters constrained
*R*[*F*^2^ > 2σ(*F*^2^)] = 0.024	*w* = 1/[σ^2^(*F*_o_^2^) + (0.0323*P*)^2^ + 2.4527*P*] where *P* = (*F*_o_^2^ + 2*F*_c_^2^)/3
*wR*(*F*^2^) = 0.060	(Δ/σ)_max_ < 0.001
*S* = 1.15	Δρ_max_ = 0.48 e Å^−^^3^
1254 reflections	Δρ_min_ = −0.31 e Å^−^^3^
73 parameters	Absolute structure: Classical Flack method preferred over Parsons because s.u. lower
0 restraints	Absolute structure parameter: 0.08 (3)
Primary atom site location: dual	

### 5,14-Diselena-3,4,15,16-tetraazatricyclo[11.3.0.02,6]hexadeca-1(13),2(6),3,15-tetraene Special details

**Table:**

Geometry. All esds (except the esd in the dihedral angle between two l.s. planes) are estimated using the full covariance matrix. The cell esds are taken into account individually in the estimation of esds in distances, angles and torsion angles; correlations between esds in cell parameters are only used when they are defined by crystal symmetry. An approximate (isotropic) treatment of cell esds is used for estimating esds involving l.s. planes.
Refinement. The H atoms were placed at calculated positions with C—H = 0.99 Å and were refined in the riding-model approximation with *U*_iso_(H) = 1.2 *U*_eq_(C).

### 5,14-Diselena-3,4,15,16-tetraazatricyclo[11.3.0.02,6]hexadeca-1(13),2(6),3,15-tetraene Fractional atomic coordinates and isotropic or equivalent isotropic displacement parameters (Å^2^)

**Table:**

	*x*	*y*	*z*	*U*_iso_*/*U*_eq_	
C1	0.4277 (4)	0.1859 (3)	0.2089 (4)	0.0163 (8)	
N2	0.4228 (4)	0.2468 (2)	0.0997 (4)	0.0186 (6)	
N3	0.2985 (5)	0.2466 (3)	0.0296 (4)	0.0206 (7)	
Se4	0.15092 (4)	0.15946 (3)	0.09906 (4)	0.02012 (13)	
C5	0.2978 (5)	0.1314 (3)	0.2288 (4)	0.0174 (8)	
C6	0.2739 (5)	0.0574 (3)	0.3352 (4)	0.0202 (8)	
H6A	0.171981	0.069125	0.380357	0.024*	
H6B	0.358169	0.063595	0.403035	0.024*	
C7	0.2750 (6)	−0.0446 (3)	0.2791 (5)	0.0250 (9)	
H7A	0.259725	−0.089763	0.353998	0.030*	
H7B	0.185083	−0.052100	0.216997	0.030*	
C8	0.4257 (6)	−0.0702 (3)	0.2058 (5)	0.0244 (9)	
H8A	0.441258	−0.024463	0.131619	0.029*	
H8B	0.413158	−0.134127	0.165772	0.029*	

### 5,14-Diselena-3,4,15,16-tetraazatricyclo[11.3.0.02,6]hexadeca-1(13),2(6),3,15-tetraene Atomic displacement parameters (Å^2^)

**Table:**

	*U*^11^	*U*^22^	*U*^33^	*U*^12^	*U*^13^	*U*^23^
C1	0.0180 (18)	0.0161 (18)	0.0147 (16)	0.0009 (14)	0.0040 (15)	0.0011 (14)
N2	0.0211 (16)	0.0173 (15)	0.0173 (14)	0.0024 (12)	0.0037 (15)	0.0004 (16)
N3	0.0230 (16)	0.0207 (17)	0.0182 (17)	0.0031 (14)	0.0039 (14)	0.0054 (15)
Se4	0.01690 (17)	0.02506 (19)	0.01841 (18)	0.00027 (17)	−0.00221 (16)	0.00498 (17)
C5	0.0170 (16)	0.0195 (18)	0.0158 (16)	0.0028 (15)	−0.0010 (14)	0.0023 (15)
C6	0.0187 (19)	0.024 (2)	0.0176 (19)	−0.0023 (16)	0.0015 (16)	0.0071 (16)
C7	0.032 (2)	0.019 (2)	0.024 (2)	−0.0069 (18)	−0.006 (2)	0.0067 (18)
C8	0.035 (3)	0.0148 (18)	0.023 (2)	−0.0014 (17)	−0.010 (2)	−0.0013 (17)

### 5,14-Diselena-3,4,15,16-tetraazatricyclo[11.3.0.02,6]hexadeca-1(13),2(6),3,15-tetraene Geometric parameters (Å, º)

**Table:**

C1—C5	1.358 (6)	C6—H6A	0.9900
C1—N2	1.388 (5)	C6—H6B	0.9900
C1—C1^i^	1.477 (8)	C7—C8	1.518 (7)
N2—N3	1.267 (5)	C7—H7A	0.9900
N3—Se4	1.885 (4)	C7—H7B	0.9900
Se4—C5	1.842 (4)	C8—C8^i^	1.541 (8)
C5—C6	1.501 (5)	C8—H8A	0.9900
C6—C7	1.540 (6)	C8—H8B	0.9900
			
C5—C1—N2	116.0 (4)	H6A—C6—H6B	107.8
C5—C1—C1^i^	126.5 (4)	C8—C7—C6	113.7 (4)
N2—C1—C1^i^	117.5 (3)	C8—C7—H7A	108.8
N3—N2—C1	117.3 (3)	C6—C7—H7A	108.8
N2—N3—Se4	110.7 (3)	C8—C7—H7B	108.8
C5—Se4—N3	86.99 (17)	C6—C7—H7B	108.8
C1—C5—C6	127.2 (4)	H7A—C7—H7B	107.7
C1—C5—Se4	109.1 (3)	C7—C8—C8^i^	114.5 (5)
C6—C5—Se4	123.7 (3)	C7—C8—H8A	108.6
C5—C6—C7	112.8 (4)	C8^i^—C8—H8A	108.6
C5—C6—H6A	109.0	C7—C8—H8B	108.6
C7—C6—H6A	109.0	C8^i^—C8—H8B	108.6
C5—C6—H6B	109.0	H8A—C8—H8B	107.6
C7—C6—H6B	109.0		
			
C5—C1—N2—N3	0.5 (5)	C1^i^—C1—C5—Se4	179.6 (3)
C1^i^—C1—N2—N3	−179.9 (3)	N3—Se4—C5—C1	0.7 (3)
C1—N2—N3—Se4	0.1 (4)	N3—Se4—C5—C6	−177.7 (4)
N2—N3—Se4—C5	−0.5 (3)	C1—C5—C6—C7	−108.5 (5)
N2—C1—C5—C6	177.5 (4)	Se4—C5—C6—C7	69.6 (5)
C1^i^—C1—C5—C6	−2.0 (7)	C5—C6—C7—C8	58.0 (5)
N2—C1—C5—Se4	−0.9 (5)	C6—C7—C8—C8^i^	63.7 (4)

Symmetry code: (i) −*x*+1, *y*, −*z*+1/2.
